# A comprehensive high cost drugs dataset from the NHS in England - An OpenSAFELY-TPP Short Data Report

**DOI:** 10.12688/wellcomeopenres.17360.1

**Published:** 2021-12-22

**Authors:** Anna Rowan, Chris Bates, William Hulme, David Evans, Simon Davy, Nicholas A Kennedy, James Galloway, Kathryn E Mansfield, Katie Bechman, Julian Matthewman, Mark Yates, Jeremy Brown, Anna Schultze, Sam Norton, Alex J. Walker, Caroline E. Morton, Krishnan Bhaskaran, Christopher T. Rentsch, Elizabeth Williamson, Richard Croker, Seb Bacon, George Hickman, Tom Ward, Amelia Green, Louis Fisher, Helen J Curtis, John Tazare, Rosalind M. Eggo, Peter Inglesby, Jonathan Cockburn, Helen I. McDonald, Rohini Mathur, Angel YS Wong, Harriet Forbes, John Parry, Frank Hester, Sam Harper, Ian J Douglas, Liam Smeeth, Laurie A Tomlinson, Charlie W Lees, Stephen Evans, Catherine Smith, Sinéad M. Langan, Amir Mehkar, Brian MacKenna, Ben Goldacre

**Affiliations:** 1 The DataLab, Nuffield Department of Primary Care Health Sciences, University of Oxford, Oxford, OX2 6GG, UK; 2 TPP, 129 Low Lane, Horsforth, Leeds, LS18 5PX, UK; 3 Department of Gastroenterology, Royal Devon & Exeter NHS Foundation Trust, Exeter, UK; 4 IBD Research Group, University of Exeter, Exeter, UK; 5 Centre of Rheumatic Diseases, King's College London, London, UK; 6 Electronic Health Records Research Group, Faculty of Epidemiology and Population Health, London School of Hygiene & Tropical Medicine, London, WC1E 7HT, UK; 7 Centre for Genomics and Experimental Medicine, University of Edinburgh, Edinburgh, UK; 8 St John's Institute of Dermatology, Guy's and St Thomas' NHS Foundation Trust, London, SE1 9RT, UK

**Keywords:** Medications, healthcare administration, biosimilars, OpenSAFELY

## Abstract

**Background:** At the outset of the COVID-19 pandemic, there was no routine comprehensive hospital medicines data from the UK available to researchers. These records can be important for many analyses including the effect of certain medicines on the risk of severe COVID-19 outcomes. With the approval of NHS England, we set out to obtain data on one specific group of medicines, “high-cost drugs” (HCD) which are typically specialist medicines for the management of long-term conditions, prescribed by hospitals to patients. Additionally, we aimed to make these data available to all approved researchers in OpenSAFELY-TPP. This report is intended to support all studies carried out in OpenSAFELY-TPP, and those elsewhere, working with this dataset or similar data.

**Methods:** Working with the North East Commissioning Support Unit and NHS Digital, we arranged for collation of a single national HCD dataset to help inform responses to the COVID-19 pandemic. The dataset was developed from payment submissions from hospitals to commissioners.

**Results: **In the financial year (FY) 2018/19 there were 2.8 million submissions for 1.1 million unique patient IDs recorded in the HCD. The average number of submissions per patient over the year was 2.6. In FY 2019/20 there were 4.0 million submissions for 1.3 million unique patient IDs. The average number of submissions per patient over the year was 3.1. Of the 21 variables in the dataset, three are now available for analysis in OpenSafely-TPP: Financial year and month of drug being dispensed; drug name; and a description of the drug dispensed.

**Conclusions: **We have described the process for sourcing a national HCD dataset, making these data available for COVID-19-related analysis through OpenSAFELY-TPP and provided information on the variables included in the dataset, data coverage and an initial descriptive analysis.

## Introduction

Medicines data can help answer important questions about the patterns of use of medications, associated costs and possible risks and benefits of pharmaceutical treatments on patient outcomes, such as death from COVID-19. In 2019–20 the NHS spent £20.9bn on medicines in England ^1^. Of this total, medicines issued in hospitals accounted for 55.9% (£11.7bn) and medicines issued in primary care accounted for 43.5% (£9.1bn). Detailed records and data exist for medicines used in hospitals; however, at the outset of the pandemic this information was not made routinely available by the NHS ^2, 3^.

Although the NHS is a single-payer healthcare system, it includes different internal payment mechanisms, and consequently different sources of medicines data. Briefly, medicines in the NHS in England are funded either centrally, by NHS England (NHSE) specialised commissioning, or locally by Clinical Commissioning Groups (CCGs). There are approximately 135 CCGs who fund all medicines prescribed in primary care, and the majority of medicines in secondary care such as those funded through overall hospital contracts or “tariffs''. However, a specific group of “high-cost” medicines, typically specialist medicines ^4^, are excluded from tariffs and funding is provided directly either from NHSE specialised commissioning or a CCG, depending on the medicine, condition or service it is used for. As a consequence, detailed payment information, including patient details, is passed between a hospital and the responsible commissioner, resulting in detailed data that is held locally by each commissioner. To our knowledge this has never been collated in a single place and made available for routine analysis at national level.

OpenSAFELY-TPP is a new secure analytics platform for electronic patient records built by our group on behalf of NHSE to deliver urgent academic and operational research during the pandemic ^5, 6^. Analyses run across all patients’ full raw pseudonymised primary care records in 40% of English general practices where TPP electronic health record (EHR) software is deployed, with patient-level linkage to various sources of secondary care data. Code and analysis are shared openly for inspection and re-use.

With the approval of NHS England, we set out to: obtain a source of hospital high-cost drug (HCD) data; make these data available in OpenSAFELY-TPP to support analysis of important questions related to COVID-19; better understand the information collected and available for analysis and generate descriptive outputs. This report is intended to support all researchers and studies carried out in OpenSAFELY-TPP, and those elsewhere working with the present dataset or similar data, to help inform the response to the COVID-19 pandemic.

## Methods

### Data source - obtaining a national high-cost drugs dataset

Hospitals in England supply medicines to patients either directly or through “ homecare” providers who deliver medicines to a patient's home. The majority of medicines are funded through overall hospital contracts, included in tariffs; however, for certain HCDs, hospitals are required to provide a submission for each patient to the relevant commissioner, either NHSE or one of 135 local CCGs ^1^, in order to receive payment. The majority of submissions relate to a prescription of a HCD, although some submissions relate to associated services (i.e. home care delivery charges). There is a national list of the medicines that are funded by NHSE ^7^ and locally agreed lists for each CCG. These patient-level submissions are processed by intermediate organisations, Commissioning Support Units (CSUs), to support financial payments and associated activities like summary reporting. To our knowledge there has been no single collation of the submissions data across NHSE and all 135 CCGs for these HCDs. To address this gap, we arranged for a single CSU, the North East Commissioning Support Unit (NECS), to collate all the data flows from their partner CSUs into a single comprehensive dataset. The data was collated by NECS in May 2020. To use the dataset in OpenSAFELY-TPP it was determined that NHS Digital must approve access, via the NHS Digital Data Access Request Service (DARs), which was granted in September 2020 and made available shortly thereafter. In line with OpenSAFELY-TPP standards on ^2^ privacy and security the HCD dataset was linked at individual patient level to primary care records in the secure data warehouse of TPP. ^3^

### Variable overview

The HCD dataset is a patient-level dataset and includes variables on patient characteristics, clinical indications and medicine prescribed ( Table 1). NHSE have a standard data collection specification for each individual submission, the Drugs Patient Level Contract Monitoring Data Set; however each CCG is independent and has local versions of the collection.

**Table 1.** Full list of variables included in the OpenSAFELY-TPP high-cost drugs dataset.

Variable name	Variable type	Specification details	Variable description
Patient_Id	n10	Mandatory where relevant	Pseudonymised patient identification, used to match dataset to other datasets within OpenSAFELY-TPP.
Financial Month	Max an2	Mandatory	Currently able to query in OpenSAFELY-TPP study definition Financial month the prescribed item was administered to patient. 1 = April; 12 = March
Financial Year	an6	Mandatory	Currently able to query in OpenSAFELY-TPP study definition Financial year the prescribed item was administered to patient. FY 2018/19 = 201819
PersonAge	n	Derived	Age of patient when prescribed item was administered to patient. Some submissions included age at intervention. Where missing this variable was derived using clinical intervention date and date of birth.
Person Gender	an1	Mandatory where relevant	Gender as stated by the patient. 1 = Male 2 = Female 9 = Indeterminate (unable to be classified as either male or female)
Activity Treatment Function Code	an3	Mandatory where relevant	Code to describe the clinical area that prescribing is taking place in, based on main speciality. Full list of codes found online ^10^.
Therapeutic Indication Code	Min an6 Max an20	Mandatory where relevant	Should be a SNOMED CT Code but looks like input varies based on organisation collecting the data. Code used to identify the reason for administering drug to the patient.
HighCost TariffExcluded DrugCode	Min an6 Max an20	Optional	Should be a SNOMED CT Code but looks like input varies based on organisation collecting the data. This should be the dm+d description of medicine administered to patient. Only populated when the provider has a dm+d enabled system.
DrugName	Max an255	Mandatory where relevant	Currently able to query in OpenSAFELY-TPP study definition Input standardisation is at a provider level rather than a national level - non-standardised text input. The name of the prescribed item. Where possible this should be the SNOMED CT name. For drugs not listed in dm+d, this must be the valid name in UPPER CASE.
RouteOf Administration	Min an6 Max an20	Mandatory where relevant	Should be a SNOMED CT code but looks like input varies based on organisation collecting the data. To be populated by providers with an e-prescribing system.
DrugStrength	Max an100	Mandatory where relevant	The amount of ingredient substance in the prescribed item.
DrugVolume	Max an100	Mandatory where relevant	The volume of the drug administered to a patient when given in liquid form.
DrugPackSize	Max an100	Optional	The amount of product in a pack or container.
DrugQuanitity OrWeight Proportion*	Max n4.	Mandatory where relevant	The quantity prescribed in terms of either the packsize or number of doses. * To note, the variable name is misspelled.
UnitOf Measurement		Mandatory where relevant	Should be a SNOMED CT code but looks like input varies based on organisation collecting the data. Describes what the DrugQuanitity Or WeightProportion variable is measuring.
Dispensing Route	an1	Mandatory where relevant	Describes where the prescription item was dispensed to the patient. 1 = Inpatient (via internal pharmacy) 2 = Outpatient (via internal pharmacy) 3 = Outsourced pharmacy 4 = Homecare delivery 5 = Community pharmacy (FP10) 6 = Other (not listed)
HomeDelivery Charge	Max n18. Max n8	Mandatory	The amount charged for delivery of items to the patient's home.
TotalCost	Max n18. Max n8	Mandatory	The total cost of the activity that includes any agreed adjustments.
Derived SNOMED FromName	Max an255	Derived by NECS	dm+d code Over 90% NULL values (see Table 2).
DerivedVTM	Max an255	Derived by NECS	dm+d code - virtual therapeutic moiety Around one third of values are NULL (see Table 2).
DerivedVTM Name	Max an255	Derived by NECS	dm+d name - virtual therapeutic moiety Around one third of values are NULL (see Table 2).

The national specification for submissions is published on the NHS Data Model and Dictionary website ^8^. A full list of the variables collected via submissions and the specification for each variable can be found on the website. Further information on the NECS data collation and standardisation processes can be found in the documentation on Github ^9^.

A description of each variable in the OpenSAFELY-TPP HCD dataset is provided below. Table 1 provides a brief overview of each variable and Table 2 provides information on the completeness of the data collection.

**Table 2.** Completeness of each variable in the national high-cost drugs dataset.

Variable Name	Records for FY 2018/19				Records for FY 2019/20			
	Total records	% missing	% Numeric (and not missing)	Number of unique values	Total Records	% missing	% Numeric (and not missing)	Number of unique values
PersonAge	2,799,394	1.8%	100.0%	114	3,984,198	2.9%	100.0%	125
PersonGender	2,799,394	0.0%	100.0%	3	3,984,198	0.0%	100.0%	4
Activity Treatment FunctionCode	2,799,394	9.2%	100.0%	144	3,984,198	6.0%	100.0%	143
Therapeutic IndicationCode	2,799,394	49.6%	12.6%	7,230	3,984,198	68.3%	36.0%	5,130
HighCostTariff Excluded DrugCode	2,799,394	29.0%	78.9%	12,687	3,984,198	43.5%	87.9%	11,722
DrugName	2,799,394	1.0%	0.0%	20,698	3,984,198	1.4%	0.1%	19,609
RouteOf Administration	2,799,394	7.3%	2.3%	1,213	3,984,198	41.8%	69.9%	625
DrugStrength	2,799,394	24.1%	11.8%	22,887	3,984,198	31.4%	13.1%	9,091
DrugVolume	2,799,394	42.2%	30.3%	12,168	3,984,198	60.8%	22.7%	7,472
DrugPackSize	2,799,394	21.8%	35.6%	7,952	3,984,198	24.7%	48.2%	7,156
DrugQuanitity OrWeight Proportion	2,799,394	6.8%	65.5%	10,349	3,984,198	7,4%	65.5%	13,871
UnitOf Measurement	2,799,394	100.0% ^1^	23.4%	57	3,984,198	77.7%	84.5%	853
Dispensing Route	2,799,394	12.4%	97.7%	235	3,984,198	24.6%	99.1%	36
HomeDelivery Charge	2,799,394	1.8%	100.0%	6,190	3,984,198	3.8%	100.0%	11,639
TotalCost	2,799,394	0.2%	100.0%	160,060	3,984,198	0.0%	100.0%	169,294
Derived SNOMED	2,799,394	92.3%	100.0%	205	3,984,198	91.7%	100.0%	218
DerivedVTM	2,799,394	30.6%	100.0%	684	3,984,198	36.5%	100.0%	737
DerivedVTM Name	2,799,394	30.6%	0.0%	682	3,984,198	36.5%	0.0%	736

^1^ Rounded to nearest decimal point - there are 657 non-missing records in total out of 2,799,394

Currently, three variables from the HCD dataset can be queried in an OpenSAFELY-TPP study: FinancialMonth, FinancialYear and DrugName. These variables are the ones most relevant to current research questions and add new information not available in other OpenSAFELY-TPP datasets. These are also some of the most complete variables in the dataset.

### Analysing the high-cost drugs dataset within OpenSAFELY-TPP

The HCD dataset covering submissions from April 2018 to March 2020 was made available to researchers within the OpenSAFELY-TPP software framework, to inform responses to the COVID-19 pandemic.

The guidance to analyse the HCD dataset via OpenSAFELY-TPP is published online and available to all ^11^. Box 1 below provides an example of the code used to include information on HCD prescriptions within an OpenSAFELY “study definition”; this code is used to define a cohort. As part of this process, users also need to create specific codelists, which cover the medications of interest. Due to the nature of the “DrugName” variable in the HCD dataset (discussed in more detail in the Results section) the codelists used to query HCD data do not follow an existing naming convention such as the British National Formulary ( BNF) or the mandated NHS standard dictionary of medicines and devices (dm+d).

**Box 1. Example of code used in an OpenSAFELY-TPP study definition to query the HCD dataset**

The example code below flags all patients who were prescribed adalimumab between October 2019 and March 2020, in the HCD dataset. The adalimumab filter is based on the adalimumab codelist, found on OpenSAFELY codelists ^13^.

prescribed_adalimumab=patients.with_high_cost_drugs(
            drug_name_matches= adalimumab_codes, 
            between = ["2019-10-01", "2020-03-31"], 
            find_first_match_in_period=True, 
            returning="binary_flag",
            return_expectations={"incidence": 0.05,}, 
        )

Further guidance on querying HCD dataset via an OpenSAFELY-TPP study definition can be found online ^11^.

- A list of the existing HCD codelists can be found on the OpenSAFELY codelists website ^12^.
- If a codelist does not already exist, then the user will need to create one. These codelists will need to be based on the unique values from the DrugName variable. A list of all the unique values for the drug name variable can be found in the analysis code under *Extended data* ^9^.

***Full variable list.*** The national HCD dataset in OpenSAFELY-TPP covers 21 variables, three of which are currently available to query via OpenSAFELY-TPP study definitions. Table 1 provides a complete list of the variables in the dataset with a brief description of the variable type and specification.

***Variable completeness.*** Variable completeness is shown in Table 2. The completeness (the percentage of records with non-missing values) differs across the variables: some variables have very few or no missing values (DrugName, PersonAge, TotalCost) whilst others are much less complete (DrugStrength, DrugVolume, TherapeuticIndicationCode). Variables that have high levels of missing data may not be suitable for inclusion in analysis and could be a target for improving the coverage of the data collection.

The number of unique values recorded in each variable is an indication of whether the variable uses nationally standardised inputs, following a codelist with restricted input at the data collection stage, or uses locally compiled lists, which will vary across providers. The DrugName variable is an example of a variable which uses locally compiled lists rather than national standardised input on collection and therefore has many unique values at a national level (>20,000). By contrast the ActivityTreatmentFunctionCode variable only has 143 unique values in the latest year, suggesting use of a nationally standardised list on collection.

### Descriptive analysis

Using OpenSAFELY-TPP, descriptive analysis of the characteristics of the patients who receive HCD can be carried out for the first time on a large scale, to inform related analysis on COVID-19. In this Data Note, we have provided some summary analysis of the demographic characteristics of patients in the HCD dataset (including age, sex, ethnicity and geographic location) and compare this patient group to other patients registered at TPP practices.

Using OpenSAFELY-TPP, we produced a descriptive analysis to better understand the demographic characteristics of patients that appear in the HCD dataset and how these patients compare to others registered at TPP practices. This analysis was restricted to patients who were registered at a TPP practice between 1st January 2020 and 31st March 2020 inclusive. Any patient who appeared in the HCD dataset between 1st October 2019 and 31st March 2020 were counted as in the HCD population, all other patients were included in the comparator population (not in HCD).

## Information governance and ethical approval

This study was approved by the Health Research Authority (REC Reference 20/LO/0651) and by the LSHTM Ethics Board (Reference 21863).

NHS England is the data controller; TPP is the data processor; and the key researchers on OpenSAFELY are acting on behalf of NHS England. This implementation of OpenSAFELY is hosted within the TPP environment which is accredited, the ISO 27001 information security standard and is NHS IG Toolkit compliant ^14, 15^; patient data has been pseudonymised for analysis and linkage using industry standard cryptographic hashing techniques; all pseudonymised datasets transmitted for linkage onto OpenSAFELY are encrypted; access, the platform is via a virtual private network (VPN) connection, restricted, a small group of researchers; the researchers hold contracts with NHS England and only access the platform, initiate database queries and statistical models; all database activity is logged; only aggregate statistical outputs leave the platform environment following best practice for anonymisation of results such as statistical disclosure control for low cell counts ^16^. The OpenSAFELY research platform adheres, the obligations of the UK General Data Protection Regulation (GDPR) and the Data Protection Act 2018. Since March 2020, the Secretary of State for Health and Social Care used powers under the UK Health Service (Control of Patient Information) Regulations 2002 (COPI), require organisations to process confidential patient information for the purposes of protecting public health, providing healthcare services, the public and monitoring and managing the COVID-19 outbreak and incidents of exposure; this sets aside the requirement for patient consent ^17^. Taken together, these provide the legal bases, link patient datasets on the OpenSAFELY platform. GP practices, from which the primary care data are obtained, are required, share relevant health information, support the public health response, the pandemic, and have been informed of the OpenSAFELY analytics platform.

### Data access and verification

Access to the underlying identifiable and potentially re-identifiable pseudonymised electronic health record data is tightly governed by various legislative and regulatory frameworks, and restricted by best practice. The data in OpenSAFELY-TPP is drawn from General Practice data across England where TPP is the Data Processor. TPP developers (CB, JC, JP, FH, and SH) initiate an automated process to create pseudonymised records in the core OpenSAFELY-TPP database, which are copies of key structured data tables in the identifiable records. These are linked onto key external data resources that have also been pseudonymised via SHA-512 one-way hashing of NHS numbers using a shared salt. DataLab developers and primary investigators (BG, LS, CEM, SB, AJW, KW, WJH, HJC, DE, PI, SD, GH, BBC, RMS, ID, KB, EJW and CTR) holding contracts with NHS England have access to the OpenSAFELY pseudonymised data tables as needed to develop the OpenSAFELY tools. These tools in turn enable researchers with OpenSAFELY Data Access Agreements to write and execute code for data management and data analysis without direct access to the underlying raw pseudonymised patient data, and to review the outputs of this code. All code for the full data management pipeline, from raw data to completed results for this analysis, and for the OpenSAFELY-TPP platform as a whole is available for review on Github.

The data management and analysis code for this paper was led by AR with contributions from WH, BMK, SD, PI and DE.

### Software and reproducibility

Data management was performed using Python, with analyses carried out using R. All of the code used for data management and analyses is openly available for inspection and re-use from the OpenSAFELY-TPP high cost drugs - research GitHub repository ( *Extended data)* ^9^. More information on data access and verification is available in the supplementary material.

## Results

### Variable overview

***Patient ID, Financial Year and Financial Month.*** The HCD dataset contains submissions from April 2018 to March 2021. In FY 2018/19, there were 2.8 million submissions for 1.1 million unique patient IDs. The average number of submissions per patient over the year was 2.6. In FY 2019/20 there were 4.0 million submissions for 1.3 million unique patient IDs. The average number of submissions per patient over the year was 3.1. However, there are only a small number of submissions for FY 2020/21, and these are prospective submissions submitted before the patient had received the medicine. We recommend that these records are ignored and not used in any analysis. The patient ID in the HCD dataset is used to match the information from this dataset to other patient-level data included in the OpenSAFELY-TPP environment. This ID allows OpenSAFELY-TPP users to include information from other data sources on the platform (e.g. hospital episodes or COVID-19 testing) in any analysis of HCD use.

The financial year and financial month variables in the HCD dataset are stored separately, which makes analysis over a specific time interval a little more complex than if it were combined as a single variable. The OpenSAFELY-TPP cohort extractor has been developed so that users can query dates easily and the translation from conventional date format to separate FY and financial month filters is done in the background of the OpenSAFELY-TPP cohort extractor.

***Drug name.*** The drug name variable is a mandatory part of the submission (where relevant) and can be used in OpenSAFELY-TPP study definitions to provide information on the HCD a patient has been prescribed in a given time period. This variable can be queried to produce: a flag to indicate a patient was ever prescribed a medicine between two dates; the first date a patient was prescribed a medicine between two dates and the last date a patient was prescribed a medicine between two dates. This variable is populated for 99% of records.

There are almost 21,000 unique values for the drug name variable in FY 2018/19 and almost 20,000 in FY 2019/20. The majority of these are not in the NHS-mandated dm+d format.

This variation in the recording of drug names in submissions means that codelists cannot be created using existing data definitions (e.g., dm+d or BNF) and bespoke codelists need to be created to try and pick up all possible variants of a drug name. These bespoke codelists are created by carrying out keyword searches on the list of unique values in the DrugName variable. The range of values in the DrugName variable is dealt with by building bespoke codelists rather than via the OpenSAFELY-TPP study definition.

Not only is there variation in how the same medicine is referenced by different providers, but we also found occurrences of misspelled drug names. For example, when constructing a bespoke code list for the medicine dupilumab, we included the misspelling dipilumab as that appeared in the DrugName variable values.

Taking the medicine adalimumab as an example, based on a keyword search, there were around 460 different ways that adalimumab was described in the DrugName variable, including various brand names ^8^. The keywords used were adalimumab, amgevita, hyrimoz, humira, idacio and imraldi. The search ignored whether letters were uppercase or lowercase. The twenty most common names that appear through this search are shown in Table 3.

**Table 3.** Top twenty names that appear in the DrugNames variable using the adalimumab keyword search as described above.

DrugName	Frequency count
ADALIMUMAB	34,498
HC ADALIMUMAB IMRALDI 40 mg Injection Pre Filled Pen	17,949
ADALIMUMAB (D2E7) - HOMECARE 40 mg Preloaded Pen	11,495
ADALIMUMAB (IMRALDI) (HOMECARE)	8,205
ADALIMUMAB (IMRALDI)	7,984
HC ADALIMUMAB HUMIRA 40 mg Injection Pre Filled Pen	7,167
Adalimumab 40mg/0.8ml solution for injection pre-filled disposable devices	6,809
ADALIMUMAB REFERENCE PRICE	4,810
HOMECARE ADALIMUMAB (IMRALDI)	4,373
ADALIMUMAB (HUMIRA)_(HOMECARE) 40 mg in 0.4mL Pre-filled Injection Pen	3,649
ADALIMUMAB(AMGEVITA)	3,005
HOMECARE IMRALDI (ADALIMUMAB)	2,926
Adalimumab	2,461
HOMECARE - ADALIMUMAB (AMGEVITA) 40 mg in 0.8ml Auto Injector Pen	2,440
ADALIMUMAB (HUMIRA) (HOMECARE)	2,251
ADALIMUMAB(HUMIRA)	1,968
Adalimumab (Homecare)	1,820
HOMECARE AMGEVITA (ADALIMUMAB)	1,703
ADALIMUMAB - IMRALDI (HOMECARE)	1,477
HOMECARE ADALIMUMAB!40mg/0.8mL! PEN (HYRIMOZ)	1,466

### Descriptive analysis

**Figure 1.**
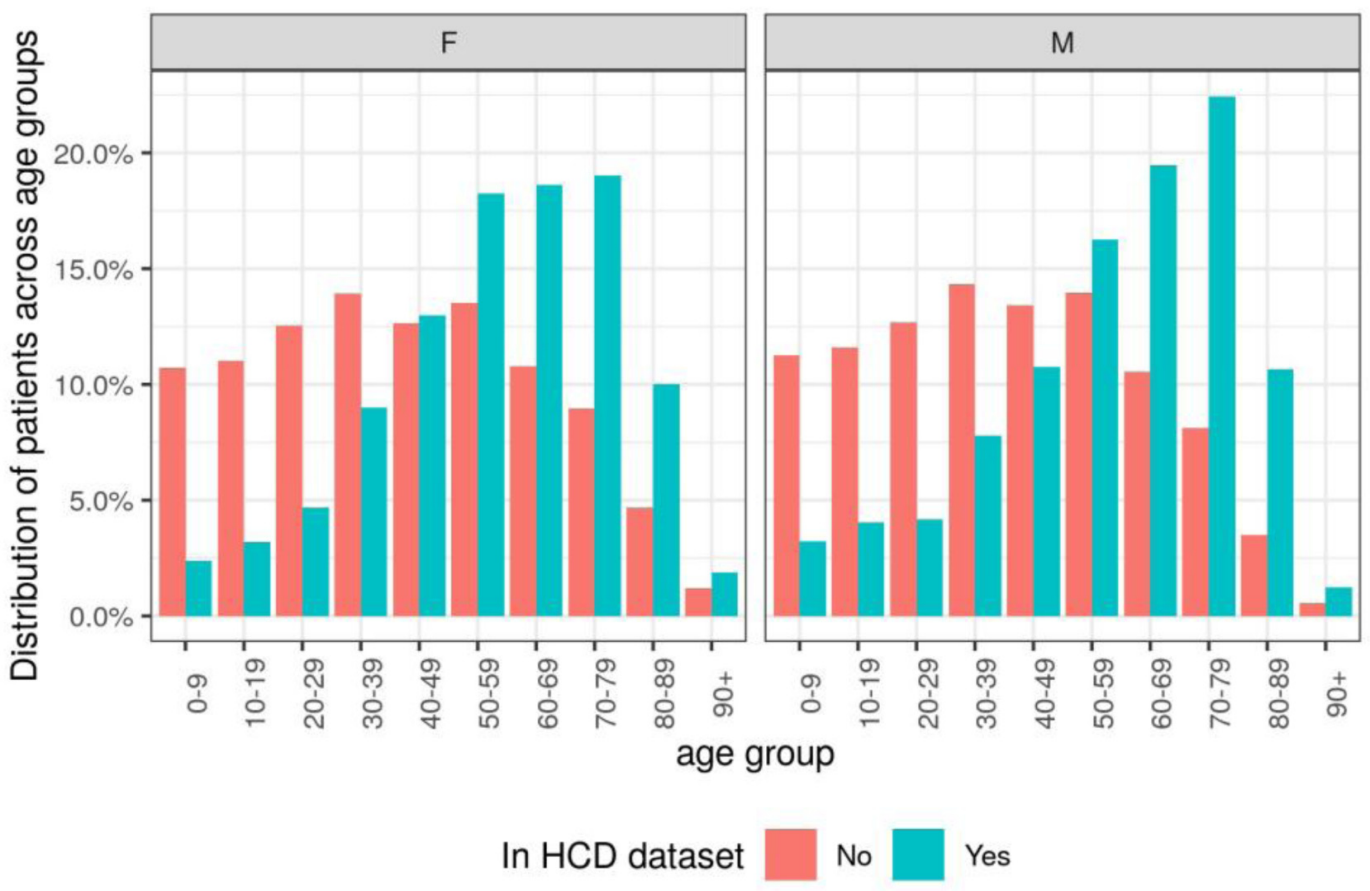
Distribution of patients across age groups, categorised by sex and whether patient appeared in the high cost drugs (HCD) dataset.

***Age.*** A higher proportion of patients in the HCD dataset belonged to older age bands (50+) than the other patients registered at TPP practices ( Figure 1, Table 4). For females, 67.8% of the patients in the HCD dataset were in older age bands compared to 39.2% of patients not in the HCD dataset. For males, 70.0% of the patients in the HCD dataset were in older age bands compared to 36.7% of patients not in the HCD dataset.

**Table 4.** Distribution of patients across age groups, categorised by sex and patient appearance in the high-cost drugs (HCD) dataset.

Age group	In HCD dataset		Not in HCD dataset	
	Number in each age group	% of total in each age group	Number in each age group	% of total in each age group
**Females**				
Total	101,596	NA	11,623,335	NA
0-9	2,437	2.4%	1,244,818	10.7%
10-19	3,235	3.2%	1,282,360	11.0%
20-29	4,736	4.7%	1,459,202	12.6%
30-39	9,125	9.0%	1,617,461	13.9%
40-49	13,200	13.0%	1,468,794	12.6%
50-59	18,547	18.3%	1,570566	13.5%
60-69	18,897	18.6%	1,251,879	10.8%
70-79	19,338	19.0%	1,042,945	9.0%
80-89	10,163	10.0%	545,253	4.7%
90+	1,918	1.9%	140,057	1.2%
**Males**				
Total	96,061	NA	11,640,791	NA
0-9	3,109	3.2%	1,311,321	11.3%
10-19	3,883	4.0%	1,348,521	11.6%
20-29	4,012	4.2%	1,474,829	12.7%
30-39	7,461	7.8%	1,669,359	14.3%
40-49	10,323	10.7%	1,563,269	13.4%
50-59	15,606	16.2%	1,624,034	14.0%
60-69	18,688	19.5%	1,229,951	10.6%
70-79	21,559	22.4%	944,582	8.1%
80-89	10,221	10.6%	408,320	3.5%
90+	1,199	1.2%	66,605	0.6%

**Figure 2.**
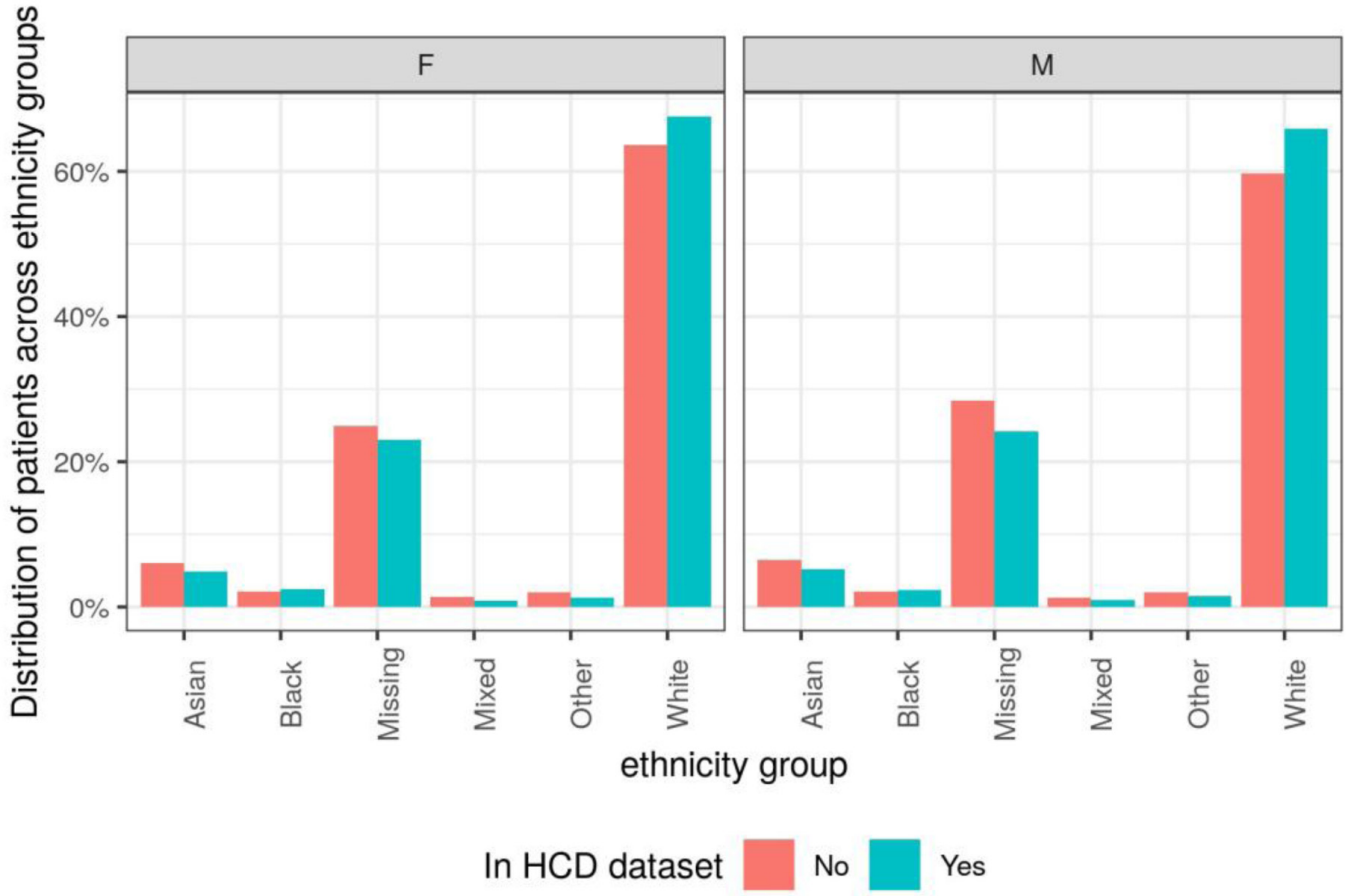
Distribution of patients across ethnicity groups, split by sex and whether patient appears in high-cost drugs (HCD) dataset.

***Ethnicity.*** A higher proportion of patients in the HCD dataset were in the White ethnicity group compared to the other patients registered at TPP practices ( Figure 2, Table 5). For females, 67.5% of the patients in the HCD dataset were in the White ethnicity group compared to 63.7% of patients not in the HCD dataset. For males, 65.9% of the patients in the HCD dataset were in the white ethnicity group compared to 59.7% of patients not in the HCD dataset.

**Table 5.** Distribution of patients across ethnicity groups, categorised by sex and whether patient appears in high-cost drugs (HCD) dataset.

Ethnicity group	In HCD dataset		Not in HCD dataset	
	Number in each age group	% of total in each age group	Number in each age group	% of total in each age group
**Females**				
Total	101,596	NA	11,623,335	NA
Asian	4,978	4.9%	700,909	6.0%
Black	2,467	2.4%	247,046	2.1%
Missing data	23,373	23.0%	2,891,214	24.9%
Mixed	897	0.9%	155,264	1.3%
Other	1,270	1.3%	228,596	2.0%
White	68,611	67.5%	7,400,306	63.7%
**Males**				
Total	96,061	NA	11,640,791	NA
Asian	4,971	5.2%	749,327	6.4%
Black	2,238	2.3%	247,208	2.1%
Missing data	23,263	24.2%	3,310,054	28.4%
Mixed	900	0.9%	150,515	1.3%
Other	1,383	1.4%	230,568	2.0%
White	63,306	65.9%	6,953,120	59.7%

***Geographical variation - STP.*** We looked at geographical variation by grouping patients by sustainability and transformation partnerships (STPs). The proportion of an STP population that appears in the HCD dataset ranged from around 0.5% to 1.5% ( Table 6).

**Table 6.** Proportion of sustainability and transformation partnerships (STP) TPP patient population that appear in the high-cost drugs dataset.

STP code	STP name	Number of patients in HCD dataset	Total patients registered at TPP practice	% of patients in HCD dataset
E54000005	West Yorkshire and Harrogate (Health and Care Partnership)	12,447	2,304,250	0.5%
E54000006	Humber, Coast and Vale	6,175	1,045,049	0.6%
E54000007	Greater Manchester Health and Social Care Partnership	2,230	219,453	1.0%
E54000008	Cheshire and Merseyside	1,787	160,579	1.1%
E54000009	South Yorkshire and Bassetlaw	8,222	1,080,379	0.8%
E54000010	Staffordshire and Stoke on Trent	766	83,909	0.9%
E54000012	Joined Up Care Derbyshire	6,139	878,428	0.7%
E54000013	Lincolnshire	9,770	659,544	1.5%
E54000014	Nottingham and Nottinghamshire Health and Care	6,434	923,839	0.7%
E54000015	Leicester, Leicestershire and Rutland	13,503	1,030,600	1.3%
E54000016	The Black Country and West Birmingham	4,510	351,433	1.3%
E54000017	Birmingham and Solihull	6,199	536,086	1.2%
E54000020	Northamptonshire	3,349	613,173	0.5%
E54000021	Cambridgeshire and Peterborough	5,257	866,038	0.6%
E54000022	Norfolk and Waveney Health and Care Partnership	12,359	870,625	1.4%
E54000023	Suffolk and North East Essex	4,580	757,888	0.6%
E54000024	Bedfordshire, Luton and Milton Keynes	6,923	1,004,396	0.7%
E54000025	Hertfordshire and West Essex	5,496	808,624	0.7%
E54000026	Mid and South Essex	13,625	1,188,216	1.1%
E54000027	North West London Health and Care Partnership	11,558	1,533,808	0.8%
E54000029	East London Health and Care Partnership	647	104,099	0.6%
E54000033	Sussex and East Surrey Health and Care Partnership	9,138	886,111	1.0%
E54000035	Surrey Heartlands Health and Care Partnership	286	42,681	0.7%
E54000036	Cornwall and the Isles of Scilly Health and Social Care Partnership	2,131	252,544	0.8%
E54000037	Devon	7,873	780,333	1.0%
E54000040	Bath and North East Somerset, Swindon and Wiltshire	9,380	909,687	1.0%
E54000041	Dorset	5,558	783,453	0.7%
E54000042	Hampshire and the Isle of Wight	4,189	596,569	0.7%
E54000043	Gloucestershire	5,455	506,331	1.1%
E54000044	Buckinghamshire, Oxfordshire and Berkshire West	236	26,080	0.9%
E54000049	Cumbria and North East	11,369	1,649,249	0.7%
NA	NA	70	8,754	0.8%

## Data validation 

### Summary

The national HCD dataset provides information on prescriptions and spend on HCD at patient level for the FY 2018/19 and 2019/20. There were 4.0 million submissions for 1.3 million unique patient IDs, with an average number of submissions per patient over the year of 3.1. This data is now available with the OpenSAFELY-TPP framework, linked to other NHS records at patient level, alongside reusable code to undertake analyses related to COVID-19. We observed substantial variation in missing data between specific fields in the data (0% gender - 77.7% unit of measurement) and consequently have made three fields available: FinancialMonth, FinancialYear and DrugName. The first output using this data in OpenSAFELY-TPP has already been published: a research paper on the association between the use of immune modifying medicines to treat immune-mediated inflammatory diseases and severe COVID-19 outcomes ^18^. This report can support those undertaking further analysis on COVID-19 using the HCD in OpenSAFELY-TPP.

### Strengths and weaknesses

The national HCD dataset includes information on all HCD, rather than being limited to a specific class of medicines or disease as some other data collections are, such as national disease registries. At the outset of the pandemic, there was no data available on medicines supplied by hospitals; the provision of HCD allows researchers and the NHS to capitalise on new information to inform analysis. We utilised an existing data collection, efficiently re-using information already collected from NHS providers, but did not add any further burden to hospitals. Access to the national HCD dataset via OpenSAFELY-TPP means that this dataset can be analysed alongside a range of other patient level information, meaning that analysis of drugs prescribed and clinical outcomes is straightforward to run, and does not involve any additional time to source data.

However, there are several caveats that need to be considered when using this resource. The national HCD dataset in OpenSAFELY-TPP is comprehensive, however due to the scale and speed at which it was assembled, it is possible that unknown inconsistencies or omissions may have occurred. The inputs to the DrugName and other variables are not standardised at a national level, which means there is a wide range of values (many thousands over a financial year). This is a feature of the data collection process. This means that the creation of bespoke codelists is required each time the HCD dataset is used for new analysis, and there can be misspellings of drug names. Alongside this non-standardised input, some variables have a high proportion of missing records. Finally, the dataset in OpenSAFELY-TPP is currently limited to a one-off collection covering submissions from FY 2018/19 and FY 2019/20.

### Findings in context

To date there has been limited research conducted using patient-level HCD data in the UK. The national HCD dataset covers all HCDs, which means that, for the first time, researchers can produce analyses covering large numbers of patients, over one million unique patients in each year. As an example, the first analysis using the HCD dataset within OpenSAFELY-TPP was conducted to ascertain the risk of severe COVID-19 outcomes associated with immune-mediated inflammatory diseases and immune modifying therapies: a nationwide cohort study in 17 million individuals ^18^. We are unaware of any other use of comprehensive and routinely collected data on medicines supplied by hospitals to individual patients in England. There are several large clinical registry studies in England focused on specific diseases or medicines; although detailed and comprehensive, they are limited by underreporting, loss to follow-up and absence of information from elsewhere in the NHS ^19– 21^. Combining detailed clinical registry data with the data available in OpenSAFELY may enhance the quality and robustness of analysis that can be achieved.

### Policy implications

In March 2020, at the outset of the COVID-19 pandemic, there was no routine comprehensive hospital medicines data from the UK available to researchers and organisations. Since the onset of the pandemic, the NHS has improved access to information on the usage of medicines in hospitals, the NHS Business Services Authority now publish a monthly summary of the volume of medicines issued in hospitals ^22^ which is publicly available and NHS Digital are developing the electronic prescribing and medicines administration (EPMA) data collection with a subset of hospital data now available ^23^. The availability of the national HCD dataset through OpenSAFELY-TPP adds to this collection of knowledge, and researchers can access this resource, along with all other OpenSAFELY-TPP data sources, by following the OpenSAFELY access process ^24^.

However, the current dataset in OpenSAFELY-TPP is a one-off collection covering submissions from FY 2018/19 and FY 2019/20, and there is no process in place as at time of writing to routinely update the information available in the HCD dataset. Whilst this is very useful for assessing events and outcomes early in the COVID-19 pandemic, a routine update of the data is needed to assess current high-priority questions and future important questions. For example, a routine update to this data will allow assessment of COVID-19 vaccine effectiveness in people using high -cost medicines or indeed people with a recorded diagnosis likely to be treated with a HCD. Our work demonstrates that it is possible for the NHS to collate the data at a national level and we strongly recommend that a routinely updated version of the HCD dataset is produced and made available to all interested users, including via the OpenSAFELY-TPP platform.

## Data availability

OpenSAFELY: https://opensafely.org/

The project contains the following underlying data:

- All data were linked, stored and analysed securely within the OpenSAFELY platform. Data include pseudonymized data such as coded diagnoses, medications and physiological parameters. No free text data are included. All code is shared openly for review and re-use under MIT open license. Detailed pseudonymised patient data is potentially re-identifiable and therefore not shared.
- For security and privacy reasons, OpenSAFELY is very different to other approaches for EHR data analysis. The platform does not give researchers unconstrained access to view large volumes of pseudonymised and disclosive patient data, either via download or via a remote desktop. Instead we have produced a series of open source tools that enable researchers to use flexible, pragmatic, but standardised approaches to process raw electronic health records data into “research ready” datasets, and to check that this has been done correctly, without needing to access the patient data directly. Using this data management framework we also generate bespoke dummy datasets. These dummy datasets are used by researchers to develop analysis code in the open, using Github. When their data management and data analysis scripts are capable of running to completion, and passing all tests in the OpenSAFELY framework, they are finally sent through to be executed against the real data inside the secure environment, using the OpenSAFELY jobs runner, inside a container using Docker, without the researcher needing access to that raw potential disclosive pseudonymised data themselves. The non-disclosive summary results output tables, logs, and graphs are then manually reviewed, as in other systems, before release.
- As part of building that resource for the community, over the next six months we are working with NHS England to cautiously on-board a small number of external pilot users to develop their analyses on OpenSAFELY. This process is described in further detail on our webpage, here: https://opensafely.org/onboarding-new-users/.

### Extended data

Analysis code available from: https://github.com/opensafely/highcostdrugs-research

Archived analysis code as at time of publication: https://doi.org/10.5281/zenodo.5747620

License: MIT
