# Supplementary material for: A comprehensive high cost drugs dataset from the NHS in England - An OpenSAFELY-TPP Short Data Report
Source: Wellcome Open Res. 2021 Dec 22;6:360. [Version 1] doi: 10.12688/wellcomeopenres.17360.1 (PMC9120928; doi:10.12688/wellcomeopenres.17360.1)
Supplement: Peer review history

# Peer review: Reviewer response for version 1

**DOI:** 10.21956/wellcomeopenres.19195.r50570

**Published:** 2022-06-06

**Authors:** Katie Page, Lutfun Hossain

**Affiliations:** 1 Centre for Health Economics Research and Evaluation, University of Technology Sydney, Sydney, NSW, Australia

This data report outlines and describes the collation of a national open-source database for high costs drugs in the NHS England. Due to complex funding arrangements, much of this information was stored locally. The aim was to produce a comprehensive large-scale database on the use of high-cost medicines to better understand the impacts of these medicines on COVID-19 outcomes. It is an ambitious but worthwhile project achieved in an impressive timeframe, and it is well described. It clearly identifies a gap in researchers’ access to detailed and reliable medicines information on high-cost drugs on a widespread scale. The authors also provide access to all code used for data management and analysis. Further efforts in this space are needed to enable researchers to conduct worthwhile studies on medicine use at a population level.  

 The dataset is comprehensive and covers the time from 2018 to 2020 (2 financial years) with over 1 million unique patients per year from 5.4 million submissions for payment for high-cost drugs. There are a total of 21 variables but only 3 are currently available for analysis in the open access data analysis platform (OpenSAFELY). Descriptive analysis shows that older (70+ years), white individuals were overrepresented in the database. A strength of the database is that the high-cost drug records can be linked to other patient records in primary and secondary care in OpenSAFELY, allowing a range of linkage studies not previously possible.

 Overall, the paper is well written and the processes for data collation are clear. There are a few things we noted:

 We would have liked a more detailed description of the range and mean costs of such drugs. What is the minimum cost of drugs to be subsidized outside of hospital contracts or tariffs? There is a brief description in the abstract but some further elaboration in the introduction would be beneficial.  Further information about the type and class of drugs in the HCD list would be useful because such details would allow international researchers a better understanding and comparison with their own countrys’ databases.

 The open access element is a real strength of the study, but we are curious as to why only three variables can be accessed in such a way and there is no cost variable so far. Are the authors proposing to add to these variables over time? The amount of missing data for total cost is low so we wondered why that was not included with the other three variables? Is this a limitation of OpenSAFELY? As health economists, including cost variables alongside clinical information, is of paramount importance.

 As noted by the authors the drug name variable, which is mandatory for reporting and has only 1% of missing data, is not in a standardized format. The researchers have provided links to access codelists that have been created for medicines in the research to date. For medicines outside of this list, researchers will need to create and use their own bespoke code or codelists to access all the data they might need. This could be burdensome or act as a barrier to use for some researchers. Nevertheless, it is a comprehensive list and with the right search tools relevant information can be extracted. Suggestions by the research team, or open source code on the most useful way to use and extract this information would be valuable.

 The dataset is just a one-off compilation and covers only 2 years’ worth of data which can be useful in the early stages of the pandemic. Updates will be essential for tracking the longer-term outcomes linked to COVID-19 including long COVID and vaccine impacts. Codelists will need to be updated on an ongoing basis to ensure all high-cost drug data can continue to be accessed.

 Overall, it is very promising that these substantial logistical and data compilation efforts have resulted in such a useful and easy to access resource. It will be interesting to see how these data are used are we look forward to seeing the outcomes of these future studies.

Are sufficient details of methods and materials provided to allow replication by others?

Yes

Is the rationale for creating the dataset(s) clearly described?

Yes

Are the datasets clearly presented in a useable and accessible format?

Partly

Are the protocols appropriate and is the work technically sound?

Yes

Reviewer Expertise:

Dr Katie Page is a senior health economist and academic psychologist with expertise in the use and evaluation of administrative health data and economic evaluations. Dr Lutfun Hossain is a pharmacist and researcher with expertise in quality use of medicines.

We confirm that we have read this submission and believe that we have an appropriate level of expertise to confirm that it is of an acceptable scientific standard.

# Peer review: Reviewer response for version 1

**DOI:** 10.21956/wellcomeopenres.19195.r50328

**Published:** 2022-05-19

**Authors:** Ruth H. Jack

**Affiliations:** 1 Centre for Academic Primary Care, Lifespan and Population Health, School of Medicine, University of Nottingham, Nottingham, UK

This data note describes the first instance of high-cost drug (HCD) prescribing data from NHS hospitals in England being collected together and made available for research. The combined dataset covers the financial years 2018/19 to 2019/20, and it is possible to link records to other datasets within OpenSAFELY in order to determine other variables, including hospital episodes and patient characteristics. Descriptions of the variables in the HCD dataset, including the number of unique values and completeness, provide a helpful guide for where improvements in data collection are needed to enable more of this dataset to be used in research.

 This is an incredibly useful piece of work, demonstrating the feasibility of collating such information but highlighting the problems when non-standard values are used. The example of drug names given, where around 460 ways of describing the drug adalimumab, shows how improvements in standardised data collection are needed, as well as open access collaboration of code lists already created. Due to the incompleteness of many of the variables, only a small proportion are of sufficient quality to be made available for research. This dataset is currently a one-off, made possible by the pressing need to understand the COVID-19 pandemic. A routinely updated dataset with more information in a useable format would be a valuable resource for understanding the fuller picture of healthcare within England.

 The processes used are clearly described, with references to publicly available codes, analysis and other resources.

 I have only a few minor revisions to suggest:

1. Adding a key to Table 1 to describe what the variable types are might be helpful.
2. The variable type is missing for UnitOfMeasurement in Table 1.
3. It would be more useful to have the number of non-missing (possibly excluding not known) records for each variable in Table 2 rather than repeating the total number of records in each financial year.
4. In 2019/20 there are four unique values for PersonGender, but only three are listed in Table 1 – what was the extra value?
5. On page 8 there are some sentences that aren’t clear: “The OpenSAFELY research platform adheres, the obligations[…]” and “GP practices, from which the primary care data are obtained, are required, share relevant health information[…]”.
6. It’s not clear where the “supplementary material” referred to in the 'Software and reproducibility' section is.
7. The column headings in Table 5 refer to age, when the table shows ethnic group data.

Are sufficient details of methods and materials provided to allow replication by others?

Yes

Is the rationale for creating the dataset(s) clearly described?

Yes

Are the datasets clearly presented in a useable and accessible format?

Yes

Are the protocols appropriate and is the work technically sound?

Yes

Reviewer Expertise:

Epidemiology using large databases of electronic health records

I confirm that I have read this submission and believe that I have an appropriate level of expertise to confirm that it is of an acceptable scientific standard.

# Peer review: Reviewer response for version 1

**DOI:** 10.21956/wellcomeopenres.19195.r50060

**Published:** 2022-05-12

**Authors:** Richard Day

**Affiliations:** 1 Department of Clinical Pharmacology & Toxicology, St Vincent's Hospital, Sydney, NSW, Australia; 2 St Vincent's Clinical Campus, University of New South Wales, Sydney, NSW, Australia

A comprehensive knowledge of medications being prescribed and dispensed across health care systems, to which people, with which characteristics, in which settings, with which outcomes and at what cost, is an important goal from many perspectives. Global and geographic trends in outcomes, costs, and access can provide critical information that can lead to better system responses. In the UK, a national, patient-level, data set of all medications used that includes those prescribed in NHS hospitals was not available for interrogation pre the COVID pandemic.

 Rowan *et al.*'s report describes the construction of a national 'high cost drugs' (HCD) data set from UK NHS, complete, patient level, hospital data. The 'pseudonymised' patient's data comprising the HCD data set were managed with the OpenSAFELY-TPP, electronic health record platform (OSP) established across a large percentage (>40%) of GP practices in England. OSP analyses in primary care access each patient's, pseudonymised, complete, electronic primary care health record data allowing patient level data-linkages to be made to secondary databases and services.

 The current exercise is designed to demonstrate to researchers the HCD data set and the availability of the 'extended' OSP resource and how this resource might be used. The case to build the extended OSP prevailed because of the COVID pandemic and the acute need for health system-wide data on impacts of multiple, new, potentially critical, medication-related interventions and consequences, often with very limited background information to guide decisions. To progress from no availability of a national data set for medicines used in NHS hospitals to a HCD data set since the start of the pandemic is a remarkable achievement especially given the overwhelming complexity of supply and funding arrangements for medications in NHS hospitals. All steps and methods in building this resource are made available for researchers to evaluate via 'open-source' access. Considerable skills and resources are likely required.

 The example discusses the characteristics of the HCD built and now ready for limited use. There were 2.8 million 'submissions' (think purchases of a high cost drug) in NHS hospitals for 1.1 unique patients for FY 2018/9 rising to 4million for 1.3 million individuals in FY 2019/20, a substantial experience to interrogate. **Why did the 'purchases' increase? **The premise behind the HCD is that it likely reflects the significant, long-term, inflammatory disease group whose outcomes, experiences and connections to secondary services and primary care could be significantly impacted by COVID. High cost drugs for these conditions are prescribed largely by specialist services in hospitals, not the community, in the UK. Three of the 21 variables in the HCD made available for the study at this preliminary stage of development show that in comparison to the remainder of the OSP cohort the HCD patients were older and more likely White. The 21 variables to describe this HCD cover information needed to understand 'high cost drug' use and outcomes associated in individual patients across the NHS hospital and primary care system in UK. For example what conditions are being treated, what 'high cost drugs' have been chosen, what dose selected, etc. Not a lot new emerged of special interest in this example, however, that was not the point. The resource and its features and how to access the resource and HCD was the goal and this has been achieved.

 In summary, an important resource produced with impressive speed, promises deeper understanding and insights around impacts of medicine use and external impacts, such as the COVID epidemic, on individual patients and the NHS is now available for interrogations. Results from these studies are eagerly anticipated.

Are sufficient details of methods and materials provided to allow replication by others?

Partly

Is the rationale for creating the dataset(s) clearly described?

Yes

Are the datasets clearly presented in a useable and accessible format?

Partly

Are the protocols appropriate and is the work technically sound?

Yes

Reviewer Expertise:

Personalising medications according to the principles of 'quality use of medicines'

I confirm that I have read this submission and believe that I have an appropriate level of expertise to confirm that it is of an acceptable scientific standard.
